# Alkaloidal Phytoconstituents for Diabetes Management: Exploring the Unrevealed Potential

**DOI:** 10.3390/molecules27185851

**Published:** 2022-09-09

**Authors:** Tapan Behl, Amit Gupta, Mohammed Albratty, Asim Najmi, Abdulkarim M. Meraya, Hassan A. Alhazmi, Md. Khalid Anwer, Saurabh Bhatia, Simona Gabriela Bungau

**Affiliations:** 1 School of Health Sciences, University of Petroleum and Energy Studies, Dehradun 248007, Uttarakhand, India; 2 Chitkara College of Pharmacy, Chitkara University, Rajpura 140401, Punjab, India; 3 Department of Pharmaceutical Chemistry and Pharmacognosy, College of Pharmacy, Jazan University, Jazan 45142, Saudi Arabia; 4 Pharmacy Practice Research Unit, Department of Clinical Pharmacy, College of Pharmacy, Jazan University, Jazan 45124, Saudi Arabia; 5 Department of Pharmaceutical Chemistry, College of Pharmacy, Jazan University, Jazan 45142, Saudi Arabia; 6 Substance Abuse and Toxicology Research Centre, Jazan University, Jazan 45142, Saudi Arabia; 7 Department of Pharmaceutics, College of Pharmacy, Prince Stattam Bin Abdulaziz University, Al-kharj 16278, Saudi Arabia; 8 Natural & Medical Sciences Research Centre, University of Nizwa, Birkat Al Mauz, Nizwa 616, Oman; 9 Department of Pharmacy, Faculty of Medicine and Pharmacy, University of Oradea, 410028 Oradea, Romania; 10 Doctoral School of Biomedical Sciences, University of Oradea, 410028 Oradea, Romania

**Keywords:** alkaloids, phytoconstituents, diabetes mellitus, diabetes complications, alternative medicine

## Abstract

The main characteristic feature of diabetes mellitus is the disturbance of carbohydrate, lipid, and protein metabolism, which results in insulin insufficiency and can also lead to insulin resistance. Both the acute and chronic diabetic cases are increasing at an exponential rate, which is also flagged by the World Health Organization (WHO) and the International Diabetes Federation (IDF). Treatment of diabetes mellitus with synthetic drugs often fails to provide desired results and limits its use to symptomatic treatment only. This has resulted in the exploration of alternative medicine, of which herbal treatment is gaining popularity these days. Owing to their safety benefits, treatment compliance, and ability to exhibit effects without disturbing internal homeostasis, research in the field of herbal and ayurvedic treatments has gained importance. Medicinal phytoconstituents include micronutrients, amino acids, proteins, mucilage, critical oils, triterpenoids, saponins, carotenoids, alkaloids, flavonoids, phenolic acids, tannins, and coumarins, which play a dynamic function in the prevention and treatment of diabetes mellitus. Alkaloids found in medicinal plants represent an intriguing potential for the inception of novel approaches to diabetes mellitus therapies. Thus, this review article highlights detailed information on alkaloidal phytoconstituents, which includes sources and structures of alkaloids along with the associated mechanism involved in the management of diabetes mellitus. From the available literature and data presented, it can be concluded that these compounds hold tremendous potential for use as monotherapies or in combination with current treatments, which can result in the development of better efficacy and safety profiles.

## 1. Introduction

Recent trends in epidemiology suggest the most common endocrine disorder is diabetes mellitus. In the US, diabetes mellitus affects over 10% of the population. The disease is expected to increase in prevalence in the coming years, affecting one in three Americans. The cure of diabetic mellitus (DM) and its consequences costs billions of dollars per year. DM is a metabolic condition in which blood glucose extent is abnormally high [1]. A person with Type 1 diabetes mellitus (T1DM) is triggered by inadequate or no insulin secretion. This causes immune-mediated cell death, requiring lifelong insulin therapy [2]. T1DM is renowned as a chronic, multifactorial disease with a strong genetic component that, through interactions with specific environmental factors, triggers the onset of this disease [3,4]. The genetic region that owns the commencement of this disease is the HLA locus. Autoimmunity conferred by humans in T1DM relies on the detection of insulitis, islet cell auto-antibodies, and activated β-cell specific T-lymphocytes [5]. The first described auto-antibodies correlated to the development of T1DM are islet cell auto-antibodies (ICA). Later, auto-antibodies to insulin (IAA), glutamic acid carboxylase (GADA), protein tyrosine phosphatase (IA2 or ICA512), and Zn (ZnT8) were also identified. Owing to the development of such auto-antibodies, the antigens released via virus-directed or any physiological mechanism facilitate β-cell death. All lead to the initiation of immune responses against other β-cells. This autoimmune response in T1DM against β-cells is possible only if the auto-reactive T-cells escape thymic negative selection. Thereafter, usually dendritic cells (DCs) carry out the function of antigen presentation to such T-cells [6].

Findings such as swelling, hypertension, insulin resistivity, insufficient insulin production, pancreatic cell collapse, and immoderate glucose release foreground DM [7]. Across the world, the incidence of gestational diabetes, type 1 diabetes mellitus and type 2 diabetes mellitus has grown in concern [8]. T2D is known to have greater risk factors because of behaviors such as sedentary lifestyle, smoking, alcohol consumption, an elevated diet, and inactivity. Inculcating a healthier lifestyle involving a diet rich in fruits and vegetables has been proposed as a prophylactic approach in T2D [9]. T1D accounts for 5% to 10% of all diabetic patients and is frequently detected in children and teen-agers. The interplay of environmental causes, such as viral infections, and genetic vulnerability leads to T1DM [10]. Unlike T2D, the considerable loss and damage of pancreatic cells cause rapid deficiency of insulin release and indications of hyperglycemia.

**Figure 1:**
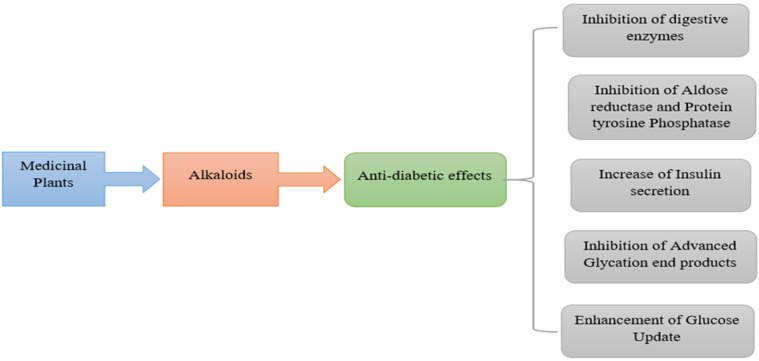
Mechanism involved in the antidiabetic activity of alkaloidal phytoconstituents.

It has now become necessary in the light of pronounced adverse repercussions caused by antihyperglycemic drugs to seek some alternatives that are as effective as present ones, while having fewer or no side effects. The mode of action that these medicines use includes inhibition of carbohydrate-digesting enzymes such as glucosidase and amylase, as well as modulation of additional key targets including insulin production and tyrosine kinase dysfunction [11]. The traditionally used herbs given as organic therapies that studies have found effective in treating human ailments contain various secondary constituents such as polyphenols and N-containing collection of phytonutrients known as alkaloids. These are found across the kingdom Plantae with a variety of biological and medicinal properties [12,13]. Other than kingdom Plantae, fungi (aquatic) and vertebrates are rich sources of such medically potent phytonutrients. The detailed description of the antidiabetic activity of alkaloidal phytoconstituents is depicted in Figure 1.

Different kinds of anti-hyperglycemic medications have been introduced during the last year. As reported by the DrugBank, more than 95% of FDA-approved antidiabetic medicines are associated with T2D by facilitating insulin secretion and thereby reducing blood glucose levels [14]. These medications are typically taken alone or in combination with supplementary antidiabetic medications. The antidiabetic synthetic drugs work by raising insulin production from the pancreas or by increasing the sensitivity towards insulin, decreasing the levels of glucose in the liver, limiting carbohydrate intake in the digestive tract, and augmenting the levels of peripheral glucose disposal. These include various medications such as acarbose, voglibose, and miglitol, which are commonly used to suppress intestinal glucosidase, although these medications have no significant impact on insulin synthesis. Sulfonylurea-based medications such as glimepiride and glyburide, on the other hand, stimulate insulin hormone production directly while having a minimal or moderate impact on enzymatic hydrolysis. Abdominal problems and diarrhea are typical adverse effects of glucosidase blockers [15]. Furthermore, thiazolidinediones have been linked to weight gain, heart failure, and anemia, among other side effects (i.e., pioglitazone and rosiglitazone). Other antidiabetic medicines have been linked to adverse side effects such as headache, hypoglycemia, dizziness, nausea, vomiting, lethargy, dyspepsia, constipation, and genital and bladder infections. Because of the probability of adverse consequences, long-term usage of orthodox medicines may be undesirable [16].

Even though contemporary antidiabetic drug design techniques have developed a new path for screening and assessing biological and synthetic molecules, no substantial achievements have been documented. The fundamental challenge with developed medications is their inability to engage with diverse sensors associated with cellular homeostasis owing to the selectivity of protein active sites for favorable substrates. According to DrugBank, alkaloids have only been used in the development of a small fraction of the anti-diabetic medications currently available (e.g., miglitol, bromocriptine, and linagliptin). As a result, in terms of exploring alternative medicines in terms of efficacy and safety, further studies to explore the molecular mechanism of these alkaloids in the management of DM may become one of the crucial components [17].

## 2. Role of Medicinal Plants in the Management of Diabetes Mellitus

Herbal medicines have been used to treat human diseases since the dawn of humanity. The anti-hyperglycemic properties of these plants were attributed to their ability to restore pancreatic cell function by enhancing insulin secretion, limiting glucose absorption in the gut, or enabling molecules in insulin-dependent activities. Medicinal herbs have been recommended by the WHO as the major source of medical care because of their ease of availability, cost, social appropriateness, and people’s faith in them [18].

Numerous herbal plants have been used in conventional healthcare systems around the world to prevent long-term difficulties in the management of DM. Out of the known medicinal plants of Indian origin with antidiabetic properties, *Ficus religosa*, belonging to the family Moraceae, has been used to prepare decoction from its bark for the treatment of diabetes. Sitosterol-d-glucoside present in its bark is found to elicit hypoglycemic effects in rabbits. Other than that, it is equipped with various bioactive compounds such as polyphenols, tannins, saponins, flavonoids, and sterols [19].

Black plum or jamun (*Eugenia ambolana*) is rich in compounds containing anthocyanins, glucoside, ellagic acid, isoquercetin, kaempferol, myricetin, and hydrolysable tannins (1-0-galloyl castalagin and casuarinin). The seeds also contain the alkaloid jambosine and the glycoside jamboline, which slow down the diastatic conversion of starch into sugar. Pterocarpus marsupium is known to have hypoglycemic, β-cell protection, and regenerative properties all because of its flavonoid content. *P*. marsupium contains terpenoids and phenolic compounds: β-sitosterol, lupenol, aurone glycosides, epicatechins, and iso-flavonoids. Of note is the insulinogenic property of Epicatechins that facilitates the enhancement of insulin release and conversion of proinsulin to insulin [20]. Although the number may be beyond counting, only a few such medicinal plants have been scientifically proven and a lot more is yet to be explored. Approximately 200 natural active molecules from herbal plants, including polyphenolic compounds, flavones, triterpenes, alkaloids, and β-sitosterol, have been separated and exhibited great antidiabetic activity through multiple mechanisms, including control of blood glucose levels and metabolic abnormalities [21].

Conventional medication is the major source of reliable treatment for over 4 billion people worldwide who live in underdeveloped nations. Pharmaceutical herbs and phytochemical components may help to delay the onset of diabetes mellitus problems. It is worth noting that each medicinal herb includes numerous phytochemicals, only a small percentage of which are therapeutically useful. Furthermore, the production of phytonutrients is affected by the plant components used, such as tree bark, leaflets, blooms, stems, fruits, and seedlings, as well as the extraction techniques used [22].

### 2.1. Alkaloids for Treatment of Diabetes Mellitus

Organic items can be either biological or man-made. These alkaloids are basic in character, contain one or more nitrogen atoms, and are usually heterocyclic and obtained from amino acids. For example, indole alkaloids are obtained from tryptophan. The extraction of alkaloids is mainly done by two methods. One is known as the Stas-Otto method, which follows the distribution of alkaloidal bases between acid or aqueous solution and immiscible organic solvent. This is performed by adding alkali to the powdered drug that facilitates alkali to mix with the acids, tannins and other phenolic substances to make it free of alkaloids. Then this mixed fraction is extracted by adding chloroform or any organic solvent using a Soxhlet apparatus. This total extract is further diluted with sulphuric acid to give an aqueous acid fraction containing alkaloid sulphate that is soluble in water. Ammonia converts the alkaloidal sulphate to ammonium sulphate, which is soluble in water. The free alkaloids, which are insoluble in water, are precipitated. The other method inculcates powdered plant material and follows the same principle to obtain a crude extract of alkaloids [23].

### 2.2. Classification of Alkaloids

**Figure 2:**
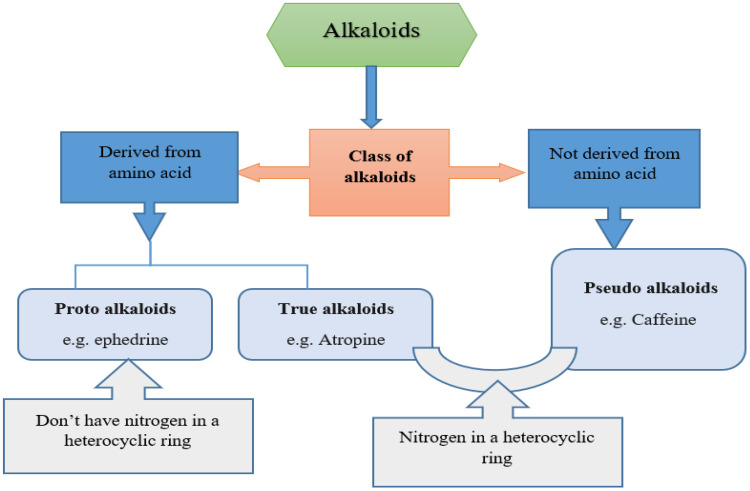
Classification of alkaloids based on chemical structures.

The classification of alkaloids is based on their pharmacological activities, chemical structure, biochemical origin, and taxonomical origin. Various alkaloids have been extracted from a variety of herbal plants and tested in various animal species for their potential antidiabetic effects. Alkaloids are mainly classified into two categories in which one class includes alkaloids derived from amino acids (proto alkaloids and true alkaloids) and the other class includes alkaloids not derived from amino acids (pseudo alkaloids). Further, the alkaloids derived from amino acids are categorized as proto alkaloids which are derived from amino acids and do not constitute a nitrogen moiety in a heterocyclic ring, e.g., ephedrine, and true alkaloids, which are derived from amino acids and have nitrogen in a heterocyclic ring, e.g., atropine. Additionally, there is a category of pseudo alkaloids that are not derived from amino acids but have nitrogen in a heterocyclic ring, e.g., caffeine. The pictorial presentation of the classification of alkaloids is shown in Figure 2.

### 2.3. Source of Various Alkaloidal Phytoconstituents Used in the Management of Diabetes Mellitus

Below is a list of phytoconstituents that have shown promising results in the management of diabetes mellitus and even diabetic complications. The data is supported by various in vitro and in vivo studies that have formed the foundation of further research in terms of clinical findings and their effects on human use. However, further studies might prove beneficial in establishing the safety and risk profile of these alkaloidal phytoconstituents. 

#### 2.3.1. Berberine

Berberine is a quinoline alkaloid extracted from *Berberis* L.’s roots and stem bark *(Berberidaceae*). *B. aristata* DC (5% in roots and 4% in stem bark), *B. asiatica* Roxb., and *B. vulgaris* are the most common sources of this chemical. *Coptis teeta Wall.* (Rhizome 8–9%) and Hydrastis *Canadensis L.* are two more species that have been observed to possess berberine. *B. sargentiana C.K. Schneid., Phellodendron amurense Rupr., and Coptis chinensis Franch* are the most common Chinese herbs. The rhizomes of *Coptis chinensis* belong to the family *Ranunculaceae* and allied species used as replacements contain around 4–8% berberine, whereas the bark of *P. amurense* has about 2–4% berberine. Berberine, which was obtained from the roots or stems of *Tinospora cordifolia* and belongs to the family *Menispermaceae*, is reported to exhibit a significant hypoglycemic effect, according to Mechanick et. al., 2020. Berberine inhibits the function of di-saccharides in Caco-2 cells, making it an antihyperglycemic drug [24]. After 72 h of pre-incubation with Caco-2 cells, it reduces sucrase activity. Berberine’s antihyperglycemic action is thought to be due to its capacity to block α-glucoside and reduce glucose transport through the intestinal epithelium. There was no notable impact on gluconeogenesis and glucose utilization in Caco-2 cells [25].

#### 2.3.2. Catharanthine, Vindoline and Vindolinine

When examined in healthy and streptozotocin-induced diabetic rat subjects, alkaloids such as catharanthine, vindoline, and vindolinine obtained from the source *Catharanthus roseus*, belonging to the family *Apocynaceae*, decreased blood sugar extent. In healthy and alloxan-induced diabetic rabbits, leeurosine, vindoline, vindolinine, and catharanthine lower blood glucose levels. Vincristine and vinblastine, dimeric alkaloids derived from the leaf and branches of *C. Roseus*, have been used to treat diabetes mellitus in several parts of the globe, notably India, the West Indies, and Nigeria [26]. Another study reported a significant anti-hyperlipidemic and antidiabetic effect of *Catharanthus roseus* leaves ethanolic extract. Since adipose tissue addresses the body’s sugar needs by sensing insulin, when nutrient intake surpasses the capacity of fat cells to store extra calories, the elevated levels of triglycerides lead to elevation of free fatty acids levels. This further adds to the hypoxic conditions in the adipose tissue. In this effect, this elevated level of free fatty acids disrupts the glucose uptake cascade driven by the combined effect of glucose concentration, insulin signaling, insulin receptor substrate-1 (IRS-1)/phosphatidylinositol kinase/kinase B (or AKT pathway) and an increased membrane localization of transporters such as GLUT4. Therefore, hypertriglyceridemia worsens the glucose metabolism, inducing subclinical inflammation and further insulin resistance and β-cell dysfunction [27]. However, ethanolic extract of *Catharanthus roseus* was administered in combination with atorvastatin, and no change in antihyperlipidemic effects was observed, whereas with an ethanolic extract of Catharanthus roseus leaves in combination with sitagliptin, a significant increase in the antidiabetic effects was observed [28].

#### 2.3.3. Calystegine B2

Griffith described a new family of nortropane polyhydroxylated calystegine alkaloids derived from the fruits of *Nicandra physalodes* L. belonging to the family *Solanaceae*, with the 3-*O*-d-glucopyranosyl calystegine B-1 structure [29]. While calystegine B1 had no inhibition activity against the enzymatic calystegine B2, which is a robust regulator of glucosidases and R-galactosidases, it did have inhibitory activity against the enzyme calystegine B2. This glucoside inhibited rice R-glucosidase effectively. The addition of glucosyl or galactosyl remainder to calystegine B2 reduced glycosidase inhibitory activity significantly. Similar results were seen with 4-o-alpha-d-galactopyranosyl-d-galactopyranose. R-glucosidase inhibitors can cure diabetes mellitus by suppressing intestinal R-glucosidases, which lower diet-induced hyperglycemic activity and internal insulin production [30]. A study was performed by Abesundara et al. to check the antidiabetic activity of the plant *Nicandra physalodes*. They used a butanolic extract of the plant in T2D by inhibiting α-glucosidase [31]. Absorption of ingested carbohydrates is delayed by an inhibitor which results in the reduction of postprandial glucose and insulin peaks. Butanolic extract of the plant is mainly used to determine the activity. The α-glucosidase enzyme is present in the brush border of the small intestine and is needed for the breakdown of carbohydrates into monosaccharides that can be absorbed. The α-Glucosidase inhibitors delay but do not stop the absorption of ingested carbohydrates, thus reducing the postprandial glucose and insulin peaks [32].

#### 2.3.4. Cryptolepine

Cryptolepine is a *Cryptolepis sanguinolenta* (Lindl.) Schltr. Indolo-quinolone alkaloid belongs to *Apocynaceae*. To explore the early architecture and biocompatibility of the cryptolepine nucleus, researchers assessed its anti-hyperglycemic properties in vitro and in a non-insulin-dependent diabetic mellitus mouse model. This study revealed the antidiabetic potential of cryptolepine, which when administered to diabetic mice, considerably reduced glucose levels. Cryptolepine’s anti-hyperglycemic influence results in a considerable drop in blood glucose levels, as well as evidence of improved insulin-mediated glucose clearance. The absorption of glucose by 3T3-L1 cells is enhanced by cryptolepine. A range of substituted and hetero-substituted cryptolepine analogs have been produced as a result of this positive association [33]. In a study carried out to explore the antidiabetic effect of *Cryptolepis sanguinolenta*, (Lindl.) Schltr. revealed its potential to reduce the intestinal absorption of glucose and thereby decrease its transport from the gut in a dose-dependent manner. Additionally, similar studies confirmed that treatment with *C. sanguinolenta* extract increases the number of β cells in experimental animals, which further improved the levels of insulin thereby decreasing the blood glucose levels in the body [34]. Various alkaloids have been identified in *C. sanguinolenta* that showed promising results as hypoglycemic agents. These alkaloidal constituents have been found to decrease the level of insulin resistance in diabetic mice induced by a high-fat diet. The mechanism involved and the antidiabetic effects were mediated by the activation of the AMP pathway, which resulted in the activation of protein kinase in 3T3-L1 adipocytes and L6 myotubes. This further led to the translocation of GLUT4 in L6 myotubes, which was found to be independent of phosphatidylinositol 3-kinase activity. Additionally, these alkaloids also improved glucose uptake pathways in an in vitro model on HepG2 and 3T3-L1 cells, which might be attributed to the decreased activity of the α-glucosidase enzyme, thereby resulting in reduction in absorption of glucose in the body. The effect mediated by *C. sanguinolenta* was not only limited to the management of diabetes mellitus but also might be effective in the prevention and treatment of diabetic complications as they decreased the levels of inflammatory cytokines, mainly IL-6, and also act as antioxidants [35].

#### 2.3.5. Harmane, Norharmane, Pinoline and Jambosine

Harmane and nor-harmane are imidazoline alkaloids obtained by *Tribulus terrestris* L., belonging to the family *Zygophyllaceae*, that enhance insulin production in the pancreatic membrane via activating imidazoline-I binding sites (3). Harmane, nor-harmane, and pinoline (carbolines) have been shown to boost insulin secretion by two- to three-fold in isolated human Langerhans islets. Harmane increases insulin production in a glucose-dependent manner [36]. A study in streptozotocin-induced diabetic rats shows a protective effect of *Tribulus terrestris* (TT) via oxidative stress inhibition more than metformin, which reduces mitochondrial reactive oxygen species (ROS) via inhibiting complex I of the electron transport cycle. Further histological examination of the liver of *Tribulus terrestris* (TT)-treated rats revealed significant liver regrowth. These results show that *Tribulus terrestris* (TT) extract might be used to treat diabetes mellitus and alleviate its symptoms in the liver [37]. Jambosine is a crystalline alkaloid present in the seeds of *Syzygium cumini,* which belongs to the Myrtaceae family. Free radicals are neutralized by jambosine, which also helps beta-pancreatic cells perform their functions better and upregulates PPARγ and PPARα [36].

#### 2.3.6. Jatrorrhizine, Magnoflorine, Palmatine, Tembetarine

Jatrorrhizine, magnoflorine, and palmatine are the three major alkaloids obtained from *Tinospora cordifolia*, which belongs to the family *Menispermaceae* [38]. These three components have been reported to possess preventive as well as curative antidiabetic properties, which were also supported by data from clinical trials. *Tinospora cordifolia* and its various formulations have been used traditionally for the treatment of diabetes mellitus. An array of studies conducted in an in vitro and in vivo animal model setting have been shown to possess promising antidiabetic results. However, due to the scattered information available regarding the mechanism involved in the management of diabetes mellitus, further studies might help in understanding the possible mechanism and combating the complex pathophysiology of diabetes mellitus. Some of the recent studies that were carried out in the past few decades revealed the presence of a few phytoconstituents shown to interact and modulate the activity of protein molecules involved in the etiology of diabetes mellitus [39]. Of these phytoconstituents, tembetarin was shown to possess potent pharmacological activity and may be considered a molecule of choice for further exploration. Further, various studies have also reported neuroactive ligand–receptor interaction as a majorly modulated pathway [39,40].

#### 2.3.7. Lepidine and Semilepidine

Seedlings of the plant *Lepidium sativum* L., belonging to the family *Cruciferae*, with an active constituent of 2-benzyl-imidazole or imidazoline were used to extract lepidine or semi-lepidine, a unique category of imidazole alkaloid [41]. Hypoglycemic effects of these chemicals were evaluated in rats induced with alloxan (toxic glucose analogue, which selectively destroys insulin-producing cells in the pancreas) for 21 days. These compounds reported substantial hypoglycemic effects. It is possible that this impact can be achieved through lowering oxidative stress and regulating enzymatic activity. Its anti-hyperglycemic activity could be mediated by stimulating pancreatic insulin production from the surviving islet cells. Furthermore, in another study, *L. sativum* methanol extract was used to treat diabetic rats, which showed decreased blood sugar and all biochemical and histological changes were restored to normal. From the above study, it could be concluded that *L. sativum* methanol extract was effective in controlling diabetes mellitus by increasing antioxidants and ameliorating the lipid profile [42].

#### 2.3.8. Mahanimbine

Mahanimbine is a carbazole alkaloid derived from *Murraya koenigii* (L.) and belongs to the family *Rutaceae.* Leaflets that are to be investigated for antidiabetic action in diabetic rats are produced by streptozotocin. Mahanimbine (i.p.) showed promising antidiabetic results in an in vivo experiment at a dose of 50 mg/kg and 100 mg/kg once a week [43]. Another study elucidated that mahanimbine exhibits anti-hyperglycemic along with anti-lipidemic effects. These results were in favor of beneficial effects of mahanimbine in the management of diabetes mellitus associated with an abnormal lipid profile and related cardiovascular complications [44]. However, the studies are only pertaining to the in vitro and in vivo animal models, which limits the establishment of further safety and efficacy profiles of *M. koenigii.* Further studies to establish the underlying mechanism involved in its antidiabetic effect might further enhance the establishment of an efficacy profile. The addition of toxicity studies might further provide scientific evidence by which to establish the risk–benefit profile of this species.

#### 2.3.9. Piperumbellactam A, B, and C

Piperumbellactam A, B, and C were isolated from the undergrowth of *Piperumbellatum* L., belonging to the family *Piperaceae*, and examined for suppression of glucosidase enzymatic activity. The alpha-glucosidase enzyme was moderately inhibited by all three alkaloids [45,46,47]. In another study, the inhibitory activity of methanolic leaf extract of *Piper umbellatum* and *Persea americana* on α-glucosidase, β-glucosidase, maltase-glucoamylase, aldose reductase, and aldehyde reductase, enzymes involved in starch digestion or diabetic complications, was checked. The results show that tested extracts strongly inhibited α-glucosidase, maltase-glucoamylase, aldose reductase, and aldehyde reductase activities. None of the extracts showed an inhibitory effect against β-glucosidase. The results of this study suggest the prospective usage of these plants to treat diabetes mellitus [47].

#### 2.3.10. Radicamines A and B

Radicamines A and B, two novel pyridinium alkaloids discovered by Shibano et. al. in the *Lobelia Chinensis Lour* family *Campanulaceae*. This herb revealed the presence of two main active constituents, radicamines A and B, which were tested for their suppressive activity against a-glucosidase. These two novel poly-hydroxy alkaloids with an aromatic ring have been demonstrated to have a biological activity that is similar to that of one-deoxy-nojirimycin, an α-glucosidase inhibitor [46]. Another study identified 208 metabolites from the *L. chinensis* species, of which 23 phytoconstituents showed promising pharmacological activities. One of the studies revealed the presence of 5-hydroxymethylfurfural, which has been shown to be effective in the management of diabetes mellitus by affecting the insulin resistance pathway and acting on various downstream pathways, including GSK3B, TNF, MAPK1, INSR, and DPP4, and thus shown to be effective in the management of T2D and various other inflammatory disorders [45].

#### 2.3.11. Schulzeines A, B, and C

The Mediterranean sponge *Penares Schulzei* yielded three novel iso-quinoline alkaloids, schulzeines A, B, and C, two amino acids and a C28 fatty acid, both of them sulfated and suppressed glucosidases [48]. Orhan analyzed the antioxidant activity of various marine organisms from Mediterranean verse in an in vitro experimental model and confirmed that *Dysidea avara* has a promising antioxidant activity. It can become one of the promising scavenging agents for free radicals. Furthermore, avarol and its byproducts have shown promising α-glucosidase enzyme inhibitory activity, which has triggered further experimentation to confirm its antidiabetic potential [49]. Similarly, various studies were carried out on Mediterranean sponge species, including *Hemimycale columella*, and reported an array of alkaloidal and various other phytoconstituents that have shown tremendous potential in DPPH (diphenyl-1-picrylhydrazy) scavenging and oxygen radical absorbance capacity (method of measuring antioxidant capacities in biological samples in vitro) [50].

#### 2.3.12. Other Sources

*Swertia chirayita Bush* family *Gentianaceae* xanthone was extracted from the n-hexane fraction and recognized as 1,8-di-hydroxy-3,5-dimethoxyxanthone (swerchirin). It has been demonstrated to have a considerable blood sugar-reducing impact in albino rats that were fasted, fed, and glucose-treated [51]. Thomson et al. investigate antidiabetic properties and insulin secretion from monolayers of BRIN-BD11 clonal pancreatic cells under the influence of aquatic bark extract of *Swertia chirayita*. In the presence of the extract, stimulated concentration-dependent insulin secretion and its enhanced action was observed in this cell line [52].

*Trigonella foenum-graecum* L. family Fabaceae is a common diabetes mellitus treatment that is especially advised for non-insulin-dependent diabetes mellitus (NIDDM) patients. Seeds have a minor and brief hypoglycemic impact in normal to moderately diabetic animals, but not in profoundly diabetic ones. Even though other putative hypoglycemic agents such as nicotinic acid are extracted from the seedlings, the hypoglycemic effect has been linked to an unidentified alkaloid designated as trigonelline [53]. Further, another study proved that the use of *Trigonella foenum-graecum* seed powder solution had positive effects in improving lipid metabolism in type II diabetic patients with no adverse effects. This study provides information that *Trigonella foenum-graecum* seed can be used as a potential therapeutic drug for the clinical management of type II diabetes mellitus [54].

The extraction of two hypoglycemic alkaloids, tecomine and tecostanine, has substantiated the traditional usage of *Tecoma stans* (L.) *Juss.*, e.g., Kunth (*Bignoniaceae*) leaf for the cure of NIDDM patients. When injected intravenously into healthy and alloxan-induced diabetic rabbits, these alkaloids had a quick hypoglycemic impact but were ineffective in pancreatectomized rabbits. Alkaloids had minimum stability and were required in such large dosages that their clinical potential was called into doubt. In Mexico, *Tecoma stans* aqueous extract (TAE) is widely used as a traditional antidiabetic remedy. Some evidence has been given for its main antidiabetic activities [55]. *Tecoma stans* L. yielded three alkaloids: boschniakine, 5-hydroxyskitanthine, and tecomine (*Bignoniaceae*). Tecomine was found to have a substantial boosting impact on basal glucose absorption in normoglycemic rat adipocytes. The other two alkaloids were inert, i.e., boschniakine and hydroxyl-skitanthine up to 100 M [56,57].

*Talinum paniculatum* Gaertner yielded through the roots three quino-lizidine alkaloids: javaberine A, J. A hexaacetate, and J. B hexaacetate, belonging to the family *Portulacaceae*. TNF, synthesized by macrophages and adipose cells, was inhibited by these alkaloids. *T. paniculatum* has also been shown to be effective as a nutritional component and in the management of diabetes mellitus [58].

*Galega officinalis* alkaloids found in Galega officinalis were extracted in high content from the plant’s leaves. Although metformin used for treating T2DM is a synthetic form of galegine, it was the toxicity caused by G. officinalis alkaloids that made its usage controversial as a hypoglycemic drug. Later it was found that even its non-alkaloid extract produces non-toxic, hypoglycemic effects well enough [59]. However, the topic of research still remains debatable as some authors have agreed to consider this as a functional food and anti-diabetic drug.

The detailed description of sources and mechanisms of action of various active constituents obtained from various natural sources is given in Table 1

## 3. Mechanism of Alkaloidal Phytoconstituents in Diabetic Complications

### 3.1. Inhibition of Digestive Enzymes

The breakdown of restorative carbohydrates by the gastrointestinal enzymatic reaction is responsible for enhancing blood sugar levels. Amylase is a hydrolytic enzyme that catalyzes the breakdown of 1,4-glycosidase bonds in starch, glucose, and a variety of oligosaccharides. Glucosidase is an enzyme hydrolase that is released along cells lining the striated boundaries of epithelial tissue in the small intestine and causes postprandial hyperglycemia by catalyzing the hydrolytic degradation of oligosaccharides into absorbable monosaccharides [61]. One of the most common ways for lowering postprandial blood sugar levels is to suppress those gastrointestinal enzymes using secondary metabolites from botanicals. Alkaloids must connect to the selective or non-competent sites of enzymes involved in processing, preventing the formation of a complex of enzyme–substrate, which decreases enzymatic actual interest in the big scheme of things [62]. A protoberberine alkaloid palmatine inhibits both alpha-amylase and alpha-glucosidase.

Recent docking studies have put forth the potential inhibitory effect of palmatine by binding the active site residues of alpha-glucosidase and alpha-amylase enzymes. Calculation of respective binding affinity and identification of the best binding pose of palmatine through these docking studies have shed light on the mechanism of its binding with these antidiabetic targets [63,64].

*Murraya koenigii* (L.) Spreng contains carbazole alkaloids such as bisgerayafoline D, bismahanimbinol, bispyrayafoline, O-methyl mahanine, O-methyl mukonal, and mahanine, which block glucosidase (IC_50_ = 38.7 0.4, 51.3%, 29.1%, 46.1 0.3%, 77.5 0.5%, and 21.4%) Mahanimbine was discovered to have substantial anti-amylase and anti-glucosidase capabilities, with IC_50_ of 83.72 ± 1.44 g/mL and 99.89 ± 1.2 g/mL, respectively. Comparably, the quinazoline alkaloids vasicine and vasicinol from *Adhatoda vasica Nees* leaf extract hinder the sucrose-hydrolyzing activity of rat intestinal-glucosidase in an extremely challenging manner, with Ki of 82 M and 183 M, and IC_50_ of 125 M and 250 M, respectively [65].

Palmatine, a protoberberine alkaloid, suppresses amylase and glucosidase functions with IC_50_ values of 1.31 ± 0.27 M and 9.39 ± 0.27 M, respectively [29]. Oriciacridone C, 1,3,5-trihydroxy-4(-dimethylallyl)-acridone and oriciacridone F were found to have a glucosidase-suppressing action (56 ± 5.4, 17 ± 1.0, and 34.05 ± 17 mM, respectively) in the branch *of Oriciopsis glaberrima*. Piperum-bellactam A, B, and C of *Piper umbellatum*, on the other hand, had a glucosidase-blocking action of 98.07 ± 0.44, 43.80 ± 0.56, and 29.64 ± 0.46 M, respectively. Employing 2-N_2_ salicylic acid and para-nitro phenyl-D gluco-pyranoside, vindo-gentianine from *Catharanthus roseus* (L.) G. inhibits amylase (IC_50_ = 74.43 ± 9.38 g/mL) and glucosidase (IC_50_ = 269.72 ± 15.44 g/mL) [66].

The steroidal alkaloids holaphylline and sarcovagine D from *Sarcococa saligna* exhibited hypoglycemic effects and alleviated diabetes-related issues in diabetic mice induced by streptozotocin (STZ). In the STZ-induced diabetic rat model, O-methylmurrayamine A and koenidine decreased blood sugar levels by 24.6 percent and 22.5 percent, respectively, during 0–300 min, equivalent to metformin (25.9%). In in vivo experiments, echinulin and arestrictin B from the root of *Combretum dolichopetalum* showed significant antidiabetic efficacy similar to glibenclamide [67].

### 3.2. Suppression of Aldose Reductase and Protein Tyrosine Phosphatase–1B

The conversion of glucose to sorbitol by aldose reductase, a key enzyme in the polyol pathway, results in an excess of reactive oxygen radicals (ROS). AR converts cytosolic glucose to sorbitol, a chemical that enters cellular walls weakly and is digested poorly. Gestational diabetes can induce sorbitol and its metabolite, fructose, to build up inside cells, causing osmotic enlargement and mobility impairment. It has a role in the detoxification of hazardous aldehydes in additional hepatocytes, the creation of fructose for semen, osmoregulatory stability within the renal tissue, and the decrease in steroids and catecholamines under normal glycemic conditions. Numerous problems appear in hyperglycemic circumstances as a result of increased polyol metabolism, which contributes to an increase in sorbitol levels and osmotic pressure, which is a link to cataract genesis. It also produces glycative stress and raises ROS by binding to receptors. AR blockers have been recommended as a way to prevent or delay such problems. Substances with antioxidant and aldose reductase-inhibiting properties in diabetes mellitus have recently piqued the science community’s interest in diabetes management [68].

Epiberberine, coptisine, and groenlandicine isolated from the rhizome of *Coptis chinensis Franch* showed the antidiabetic activity with the IC 50 of 100.07 ± 0.63, 118.36 ± 0.78, and 140.13 ± 6.50 µM for rat lens AR, respectively, and 168.10 ± 05.51, 187.27 ± 10.03, and 154.19 ± 07.17 µM for human recombinant AR, respectively (61). AR-inhibiting effects are mediated through the dioxymethylene structure and its oxidized state inside the D and A rings of proto-berberine-type alkaloids [69].

Nevertheless, alkaloids, such as berberine chloride, berberine sulfate, berberine iodide, palmatine sulphate, and palmatine iodide obtained from the roots of *Coptis japonica*, showed a suppressive activity against aldose reductase with IC_50_ values of 13.98, 13.45, 32.84, 51.78, and 51.78 nM, respectively. Isoquinoline alkaloids from *Tinospora cordifolia* stem, *Jatrorrhizine, palmatine,* and magnoflorine, suppressed male Wistar rats lens aldose reductase with IC_50_ values of 3.23, 3.45, and 1.25M, respectively [70].

Protein tyrosine phosphatase-1B (PTP-1B) is engaged in a variety of indicating pathways and is available in musculoskeletal and hepatic fat cells as well as the central nervous system. Because it acts as a bad controller of insulin and leptin signaling, inhibiting its function, it helps in the treatment of DM and accompanying headaches. Suppression of PTP-1B promotes a rise in glucose absorption by increasing the phosphorylation of the insulin receptor and insulin receptor substrates 1 and 2 [71].

Cathinone alkaloids, such as picrasidine L, 3,4-dimethyl-canthin-5,6-dione, 4-ethyl-3-methylcanthin-5, 6-dione, eurycomine E, 5-methoxy-canthin-6-1, and 5-acethoxy-canthin-6-1, inhibit PTP-1B’s ability to carry out its normal activities with IC_50_ values of 19.80 ± 0.62, 24.72 ± 0.26, 27.83 ± 0.68, 19.18 ± 0. 76, 20.30 ± 0.24, and 28.89 ± 0.52 µM, respectively, utilizing p-nitrophenyl phosphate as the substrate. Picrasidine L inhibited PTP-1B in a competitive mode, whereas relaxing revealed a noncompetitive mode. Through a substrate-based approach, vindogentianine from *Catharanthus roseus* (L.) G. Don inhibits PTP-1B with an IC_50_ value of 15.28 ± 2.59 M [72].

Proto-berberine alkaloids berberine, coptisine, and epi-berberine and the aporphine alkaloid magno-florine extracted by *Coptis chinensis Franch* demonstrated significant suppressive activity toward PTP-1B, with IC_50_ levels of 16.43, 51.04, 24.19, and 28.14 M, respectively. Through the Lineweaver-Burk and Dixon plots, berberine and epiberberine exhibited a blended kind of suppression, whereas magnoflorine and coptisine exhibited a non-competitive type of suppression, in contrast to PTP-1B. *Nigella glandulifera Freyn* seeds contain nordi-terpenoid alkaloids, nigelladine A, B, and C, which suppress the PTP1B enzyme [73].

### 3.3. Enhancement of Insulin Release

The antipathetic effects of glucagon and insulin secreted by the tissues of the pancreatic islets of Langerhans on peripheral organs allow blood sugar levels to be managed. Insulin lowers blood sugar levels via boosting sugar absorption in muscle, lowering liver glucose production, and slowing lipolysis. Glucagon, on the other hand, raises blood glucose levels via boosting glucose production and lipid metabolism [74].

In healthy people, the incretin hormonal glucagon-like peptide-1 (GLP-1) and glucose-based insulin tropic polypeptide (GIP) promote insulin secretion in answer to meal intake. GLP-1 generated L-cells of the upper gastrointestinal tract by the proglucagon gene, which enhances insulin biosynthesis and exudation, lowers glucagon levels, diminishes appetite, lowers glucagon discharge, slows stomach exudation, and enhances pancreatic islet-cell rejuvenation and differentiation in response to meals. GIP is a glycoprotein released by K cells (gip gene) in the upper gastrointestinal tract that aids glucose digestion by increasing insulin release [75].

It is also involved in fat cell metabolism, boosts lipid–protein activity, regulates fatty acid production, and increases cellular growth and preservation. GLP-1 and GIP have very short half-lives due to DPP-IV migration, ranging from 1 to 2 min for GLP-1 to 4 min for GIP. Leading to problems in the uptake of GLP-1 and GIP, suppressing DPP-4 promotes the balance of insulin and glucagon. Various studies have discovered that inhibiting DPP-4 causes an increase in cellular ability, body structure, and mass via incretin production, which affects the continual entry of insulin after meals to lower glucose levels [76].

Lupanine, 13-hydroxy-lupanine, and 17-oxo-lupanine are quino-lizidine alkaloids derived from Lupinus species that can stimulate insulin release in a glucose-dependent manner. Lupanine enhances insulin production by inhibiting ATP-sensitive potassium channels and increasing the expression of insulin-releasing genes. Trigonelline derived from *Trigonella foenum graecum* and *Mirabilis jalapa* L. shows antidiabetic behavior by causing insulin resistance to increase. Furthermore, palmatine and berberine demonstrated antidiabetic efficacy by inhibiting DPP-4 with IC_50_ values of 8.7, 1.82 and 13.3 µg/mL, respectively [77].

### 3.4. Inhibition of Advanced Glycation End Products

The development of advanced glycation end products (AGEs) is caused by the response between C=O groups in reducing glucose and amino groups of proteins, nucleic acids, and lipids. AGEs are responsible for cell breakdown in diabetes mellitus (retinal detachments, nephropathy, nerve damage, and congestive heart failure), as well as coronary artery disease and growing older, by altering gene transcription, intracellular indicators, the release of proinflammatory molecules, and the production of reactive oxygen species (ROS) [78]. Through an in vitro bovine serum albumin (BSA)/methylglyoxal reagent (MGO) assay, 9%, 83.3%, 26.1%, and 98.2%, respectively, at 150 M. The catechol organization and a few electrons at N2 position are what make these benzylisoquinoline alkaloids interesting. Berberine (the major ingredient of *Rhizoma coptidis*) has also been shown to prevent glycosylation and have antioxidant properties. Likewise, leonurine, a natural alkaloid derived from *Herba leonuri*, has been shown to suppress massive AGEs. The capabilities of matrine-type alkaloids in the regulation of DM and associated problems were verified by Liu et al. [79]. As a final note, natural products prevent AGEs through a variety of mechanisms, including product decomposition, amino acid protection, the elimination or reduction of reactive C=O groups, and the regulation of reactive oxygen species (ROS) [80].

### 3.5. Enhancement of Glucose Uptake

By permitting glucose transporter 4 (GLUT-4) to translocate, extreme efforts aid in the management of diabetes mellitus. Various alkaloids from medicinal flora have been reported to increase glucose absorption. In correlation to vindoline I, II, and IVIII from *Catharanthus roseus* (L.), G. Don has been implicated in glucose uptake in TC6 and C2C12 cells as well as the carotenoid uptake, trying to prove to be beneficial in high blood sugar. Likewise, the identical plant’s vindogentianine causes a significant increase in glucose absorption in TC6 pancreas and C2C12 muscle tissue [81]. Alkaloids stimulate the GLUT-transporter’s translocation by performing allosteric activation of AMP-activated protein kinase. This kinase is known as the cellular fuel sensor and glucose transporter regulator. It stimulates glucose uptake and modulates insulin secretion. A study has suggested alkaloids stimulate the GLUT4-translocation via activation of the AKT (Ser473)-dependent signaling pathway [82].

**Figure 3:**
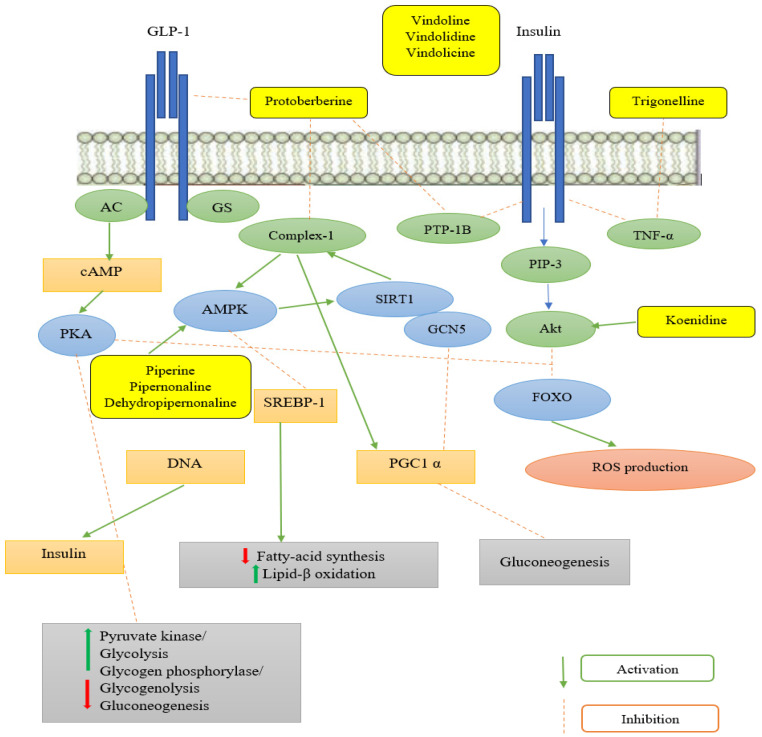
Regulation and management of diabetes mellitus through cellular signaling mediated by alkaloidal phytoconstituents.

**Figure 4:**
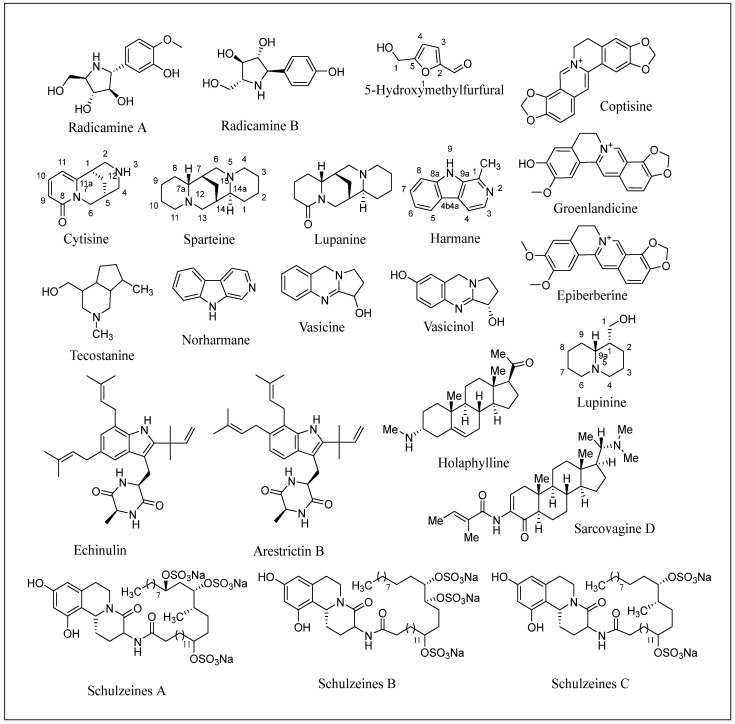
Chemical structures of various alkaloidal phytoconstituents obtained from various natural and synthetic sources.

Carbazole alkaloids, especially eight-biskoenigine, koenimbine, O-methylmurrayamine A, koenidine, mahanimbine, and murrayazoline, isolated from *Murraya koenigii* (L.) Spreng, demonstrated 1.41, 0.04, and 1.34 zero glucose absorption capacity in L6-GLUT4 myc myotubes at approximately 25 M, 13-fold; 1.42 M, 0.04-fold; and 1.26 M, 0.02-fold, respectively. Tecomine from *Tecoma stans* stimulates glucose absorption in rat adipocytes (EC_50_ = 6.79 109 M). In addition to their antidiabetic properties, alkaloids have been shown to be effective in the treatment of neurological diseases, inflammatory intestinal diseases, infectious diseases, microbial infections, malignancies, and other diseases. Numerous investigations into medicinal plants have been conducted in recent years to uncover bioactive chemicals. The detailed presentation of phytoconstituents involved in the regulation of insulin uptake and utilization is depicted in Figure 3 and structures of these compounds are presented in Figure 4.

Diabetes mellitus is an endocrine metabolic condition characterized by high blood sugar levels, insulin resistance, and ineffective insulin secretion. It has a detrimental impact on human health and can lead to more serious complications such as atrophy of the liver, brain, kidneys, and eyes. Current diabetes medications include a number of side effects that can lead to insulin resistance and financial difficulty. A comprehensive investigation by researchers revealed that medicinal plants containing phytochemicals have effective antidiabetic properties, especially alkaloids, which are heterocyclic organic molecules containing nitrogen with fewer negative effects and multiple mechanisms of action. There are various types of alkaloids, but indole, isoquinoline, amino, and terpenoidal alkaloids have been found to have an antidiabetic effect. These alkaloids regulate the insulin activity either by provoking or retarding the expression of multiple molecules such as AMP-activated protein kinase (AMPK), sterol regulatory element-binding proteins 1, glucokinase (GK), acetyl-CoA carboxylase, glucose transporter-4, glucose-6-phosphatase (G-6Pase), glycogen synthase kinase-3, protein tyrosine phosphatase 1B (PTP1B), and peroxisome proliferator-activated receptor (PPAR-γ). Isoquinoline alkaloids such as protoberberines inhibit AMPK, PTP1B, and DPP-IV, boosting glucose absorption and expressing more insulin receptors. In this class, trigonelline raises the GK, G6Pase ratio in the liver and lowers TNF-alpha levels, thus maintaining the levels of glucose and lipids. Indole alkaloids such as vindoline, vindolidine, and vindolicine inhibit α-amylase and α-glucosidase activity, which thereby decreases blood glucose levels by increasing insulin activity. Similarly, koenidine is a physiologically stable alkaloid that lowers blood glucose levels after meals and improves insulin levels. Harmine, a key alkaloid in this family, helps β-cells proliferate and differentiate, thus increasing insulin expression. To manage diabetes mellitus, aegeline, an amino alkaloid, enhances GLUT-4 translocation, boosts intracellular Ca^2+^ signalling, and modulates different cellular processes. Piperidine alkaloids manage diabetes mellitus by increasing basal glucose absorption, lowering blood glucose levels, potentiating pancreatic insulin production, and activating AMPK and PPAR-γ, thereby controlling T2D.

## 4. Future Perspectives

The current therapeutic regimen for the treatment of diabetes mellitus is mainly restricted to the use of synthetic or allopathic antidiabetic medications. Although these therapies provide symptomatic relief, they are associated with various side effects which include weight again, hepatitis, cardiovascular risk, gastrointestinal disorders, severe hypoglycemia, and various others. These side effects thus limit the use of allopathic medication and are often seen with non-compliance to the treatment as well. Due to these issues, current research has been inclined to explore various other therapeutic regimens which not only aim to provide promising results but are also associated with fewer or lesser side effects. Over the past few decades, the field of naturopathy has seen an exponential increase in the demand for antidiabetic medications, which has provided promising results not only in the management of diabetes mellitus but diabetic complications as well. Phyto-chemicals are isolated from various natural sources which include plants, algae, and fungi, and their chemical leads along with their analogs are now chemically synthesized having similar or enhanced pharmacological properties. The science of drug design has opened avenues to chemical synthesis of the same in-class or sister compounds which have better pharmacokinetic and pharmacodynamic properties. Metformin, one of the most commonly used antidiabetic drugs, is now derived from *Galegine officinalis* and is widely used in the treatment of T2D. Similarly, various phytochemicals which include flavonoids such as quercetin, alkaloids such as berberine, terpenes such as thymoquinone, and phenylpropanoids such as chlorogenic acid are used for their anti-diabetic properties. These phytochemicals show similar mechanisms of action, which are mediated through DPPH-4 activity by inhibiting α-glucosidase enzyme activity, inhibition of α-amylase, and increasing glucose uptake by adipose and muscle cells of the body. These phytochemicals have shown promising results in various in vitro and in vivo experiments; however, due to limited data available on the toxicity profile of these components, the further establishment of risk–benefit ratios is deemed necessary. The use of these phytochemicals is not only limited to Ayurveda but is established in Unani and Chinese herbal medications as well. Thus, further studies to enhance the safety and efficacy profile of these active constituents obtained from natural sources might overcome the hurdles associated with synthetic or allopathic medicine.

## 5. Conclusions

Phytoconstituents are often utilized to treat diabetes mellitus since they are readily obtainable and often inexpensive. The usefulness of phytochemicals in the control of diabetes mellitus has been continuously proven by experimental confirmations of different plant species with different mechanism pathways. Furthermore, many herbal medications which are under consideration still require attention by scientists, and only a handful of them have the ability to induce serious adverse consequences or significant drug–drug interactions. As a result, there is an unmet need to discover and develop new poly-herbal formulations and nutraceuticals derived from biological resources, particularly those containing unadulterated phytonutrients, for cure and treatment of diabetes mellitus and its major comorbidities.

Plant-based natural products are now widely used in the treatment of a variety of infectious diseases. Natural products have distinct metabolites, particularly alkaloids that work distinctively against infections and enable healthcare solutions to be delivered with fewer side effects. Even though multiple in vitro and in vivo experiments have demonstrated that alkaloids are excellent phytoconstituents, the biocompatibility of both the phytoconstituent alone and in a combination that provides synergistic outcomes must be adequately assessed. Alkaloids with various functions in diabetes mellitus control should be further investigated to maximize their potential as an antidiabetic medication or nutritional enhancer.

## Figures and Tables

**Table 1:** Source and nature of alkaloidal phytoconstituents used for the management of diabetes mellitus [2].

Phytoconstituent (Alkaloids) Source	Chemical Structure	Mechanism of action
Avarol *Dysidea avara*	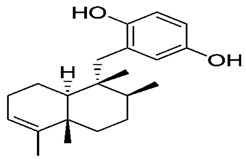	Inhibition of α-glucosidase enzyme can help in delaying digestion of carbohydrates, thereby reducing the levels of glucose in blood [49]. IC_50_ value for various avarol derivatives: 0.05–0.12 mM
Berberine *Berberis* spp. *Tinospora cordifolia*	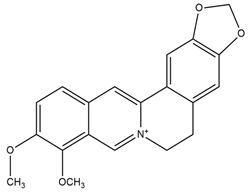	Berberine is known as an AMP-activated protein kinase (AMPK) activator. Its insulin-independent hypoglycemic effect is related to inhibition of mitochondrial function, stimulation of glycolysis and activation of AMPK pathway, which inhibits alpha-glucosidase [25]. IC_50_ value: 0.68 μM
Casuarine 6-o-a-glucoside *Syzygium malaccense*	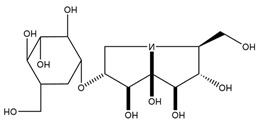	It acts by stimulating insulin secretion, inhibiting intestinal α-amylase activity, and increasing muscle basal glucose uptake along with antioxidant activity [60]. IC_50_ value of casuarine compounds: 9.7 μM
Catharanthine, Vindoline, and Vindolinine *Catharanthus roseus*	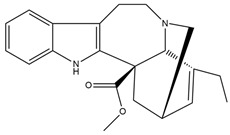 Catharanthine 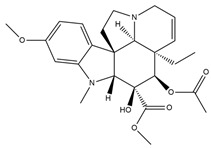 Vindoline 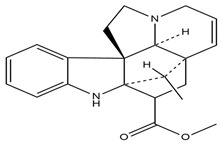 Vindolinine	Vindoline exhibits an insulinotropic effect by enhancing glucose-stimulated insulin secretion (GSIS). It was also found to increase plasma insulin in STZ-induced diabetic rats. In a recent study, vindoline reduced the voltage-dependent outward potassium currents through Kv2.1 inhibition. The combined effects resulted in fasting plasma glucose, improved oral glucose tolerance, and lowered serum glycated hemoglobin (HbA1c) and triglyceride (TG) levels [26]. IC _50_ values: 59.6 μM, >30 µM, and >50 μg/mL, respectively.
Calystegine B2 *Nicandra physalodes*	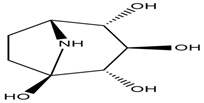	In an in vitro study, calystegine B2 inhibited mainly sucrose activity by β-glucosidase alpha inhibitor and intestinal glucose absorption [30]. IC_50_ value: range 4.6 µM
Cryptolepine *Cryptolepis sanguinolenta*	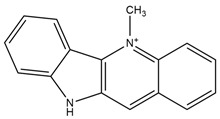	Exhibits similar effects as of glibenclamide by inhibiting the ATP-sensitive potassium channels inhibitory regulatory subunit sulfonylurea receptor 1 and by activating AMP-activated protein kinase [33]. IC_50_ value of cryptoleptine derivatives: 27–41 μM
Harmane, Norharmane, Pinoline *Tribulus terrestris*	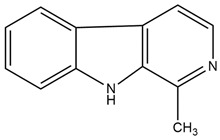 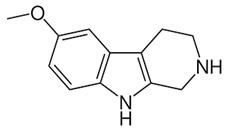 Ponoline	Stimulatory action on insulin secretion by the activation of imidazoline-I binding sites in the pancreatic cell [36]. IC_50_ values: 5 μM; 51–58 μM and 0.11 μM
Jambosine *Syzygium cumini*	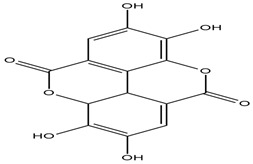	Reduces free radicals, improves the functioning of beta-pancreatic cells, and upregulates the PPARγ and PPARα [36]. IC_50_ value: 2.5 nM
Jatrorrhizine, Magnoflorine, Palmatine, Tembetarine *Tinospora cordifolia*	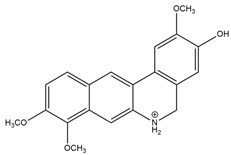 Jatrorrhizine 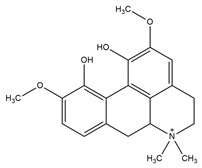 Magnoflorine 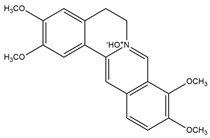 Palmitine 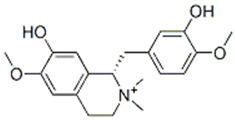 Tembetarine	Lowering of blood glucose, increase in insulin sensitivity, inhibition of a-amylase and a-glucosidase activities, direct effect on carbohydrate metabolism [39,40]. IC_50_ value (derivatives): ~1.05 μM
Javaberine A, Javaberine B *Talinum paniculatum*	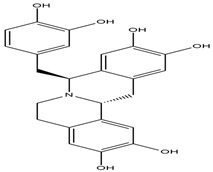 Javaberine A 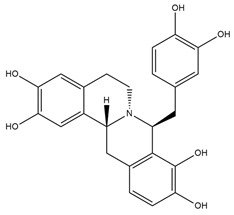 Javaberine B	Very limited data is available for these molecules; however, inhibitory activity in an in-vitro setting was noted by inhibition of TNF-α [39,40]. IC_50_ value: 23.5 µg/mL
Lepidine and semilepidine *Lepidium sativum*	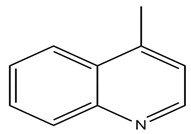 Lepidine 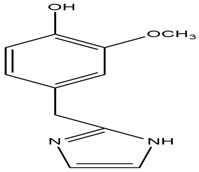 Semilepidine	Reduction in oxidative damage and modulation of antioxidant enzymes, potentiation of pancreatic secretion of insulin from the remaining islet β cells [42]. IC_50_ value: 1.42 ± 0.04 mg/mL
Mahanimbine *Murraya koenigii*	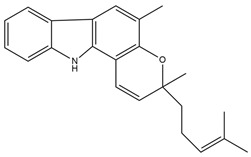	Inhibits alpha-amylase and alpha-glucosidase [44]. IC_50_ value: ranges from 3.5 to 64 μM
Piperumbellactam A *Piper umbellatum*	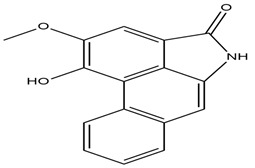	Inhibits α -glucosidase, maltase-glucoamylase, aldose reductase, and aldehyde reductase [45]. IC_50_ value of derivatives: Range 29.64 ± 0.46–98.07 ± 0.44
Swerchirin *Swertia chirayita*	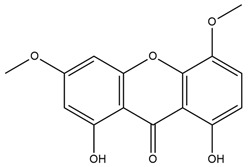	Lowers blood glucose level by stimulating insulin release from islets of Langerhans [51]. IC_50_ value: 20 μM
Radicamine A, B *Lobelia Chinensis Lour*	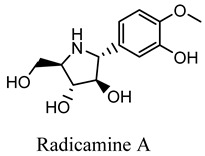 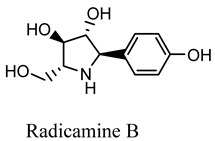	Suppressive activity against a glucosidase [46]. IC_50_ value: 54.6 µg/mL
Schulzeines A, B, and C *Penares Schulzei*	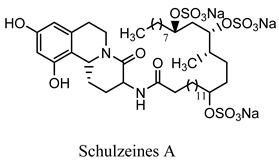 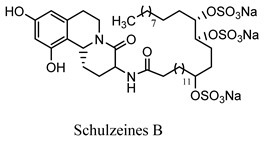 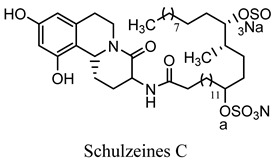	Inhibitors of glucosidase activity [48]. IC_50_ value range: 48−170 nM
Trigonelline *Trigonella foenum-graecum*	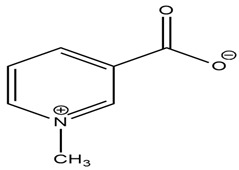	Glucose transport, carbohydrate digestion, and absorption [53]. IC_50_ value: 233 ± 0.12 µM
Tecomine Tecostanine *Tecoma stans*	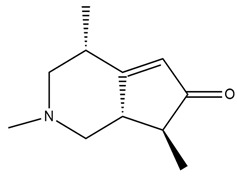 Tecomine 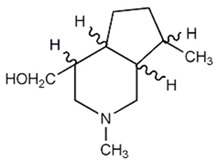 Tecostanine	Boosting effect on glucose absorption [57]. IC_50_ value: Not available

## Data Availability

Not applicable.

## Abbreviations

AMPK	AMP-activated protein kinase
DM	Diabetes mellitus
DPP-4	Dipeptidyl peptidase-4
G-6Pase	Glucose-6-phosphatase
GIP	Glucose-based insulin tropic polypeptide
GLP-1	Glucagon-like peptide-1
GLUT-4	Glucose transporter type 4
GSK3B	Glycogen synthase kinase-3 beta
INSR	Insulin receptor
MAPK1	Mitogen-activated protein kinase 1
NIDDM	Non-insulin-dependent diabetes mellitus
PTP1B	Protein tyrosine phosphatase1B
T1D	Type 1 diabetes
T2D	Type 2 diabetes
TNF	Tumor necrosis factor
WHO	World Health Organization
